# Treating Gingival Recessions Using Coronally Advanced Flap or Tunnel Techniques with Autografts or Polymeric Substitutes: A Systematic Review and Meta-Analysis

**DOI:** 10.3390/polym14071453

**Published:** 2022-04-02

**Authors:** Manuel Toledano-Osorio, Esther Muñoz-Soto, Manuel Toledano, Marta Vallecillo-Rivas, Cristina Vallecillo, Pablo Ramos-García, Raquel Osorio

**Affiliations:** Faculty of Dentistry, Colegio Máximo de Cartuja s/n, University of Granada, 18071 Granada, Spain; mtoledano@ugr.es (M.T.-O.); emsoto@ugr.es (E.M.-S.); mvallecillo@correo.ugr.es (M.V.-R.); cvallecillorivas@hotmail.com (C.V.); pramos@correo.ugr.es (P.R.-G.); rosorio@ugr.es (R.O.)

**Keywords:** connective tissue, coronal advanced flap, gingival recession, guided periodontal tissue regeneration, surgical flaps, systematic review, tunnel technique

## Abstract

Gingival recessions are a prevalent oral mucosa alteration. To solve this pathology, palatal mucosa or polymeric soft tissue substitutes are used when performing coronal advanced flap (CAF) or tunnel (TUN) surgical techniques. To evaluate which is the most successful approach, a literature review and meta-analysis were conducted. For the electronic search the National Library of Medicine, the Cochrane Oral Health Group Trials Register, EMBASE and WOS were used. Pooled data for the percentage of root coverage was collected and weighted means were calculated. Heterogeneity was determined using the Higgins (I^2^) statistic and a random-effects model was applied. Thirteen studies were included in the systematic review (12 randomized and 1 controlled clinical trials) in which both techniques (394 patients) were compared with a follow-up of 4 to 12 months. Galbraith and Baujat plots were used to control for the presence of potential outliers. After performing the meta-analysis (11 studies), the mean root coverage was similar when using the TUN or CAF techniques (*p* = 0.49). The only differences between the two were found for single recessions, where CAF offered a higher percentage of root coverage (mean difference = 4.98%; *p* = 0.006). There were no differences when applying an autograft or a polymeric substitute with either of the two tested surgical techniques (*p* = 0.445).

## 1. Introduction

Gingival recession (GR) is defined as the apical migration of the gingival margin surpassing the cement-enamel junction (CEJ). This may cause the exposure of the root surface to the oral environment [1,2]. The high incidence of this defect, approximately 54% and 100% in young adults and middle-aged adults, respectively, can be attributed to a complex pathophysiology divided into direct causes and predisposing factors [3,4]. Chronic inflammatory periodontal disease, occlusal trauma, chronic trauma, especially aggressive tooth brushing, and periodontal treatment can cause this condition [3,5,6]. Moreover, some factors, such as tooth anatomy and position, decreased alveolar bone crest thickness, bone dehiscence, soft tissue thickness, muscle traction, frenulum insertion or orthodontic treatment, favor its development [3,5,6].

GRs are normally associated with the loss of periodontal tissues [7]. Recently, the term “gingival recession” has begun to be replaced by “periodontal recession”, which is considered to represent a more precise definition of this pathology [7]. As a result of the presence of periodontal recession, dentin hypersensitivity may occur. Root surfaces are exposed to the oral environment and may develop carious and non-carious cervical lesions [8]. In addition, these alterations are esthetically unacceptable for many patients [8]. Therefore, the indications for the treatment of gingival recessions may be associated with esthetic requirements, inadequate gingival width, root hypersensitivity and difficulty for optimal hygiene [9,10,11].

**Figure 1:**
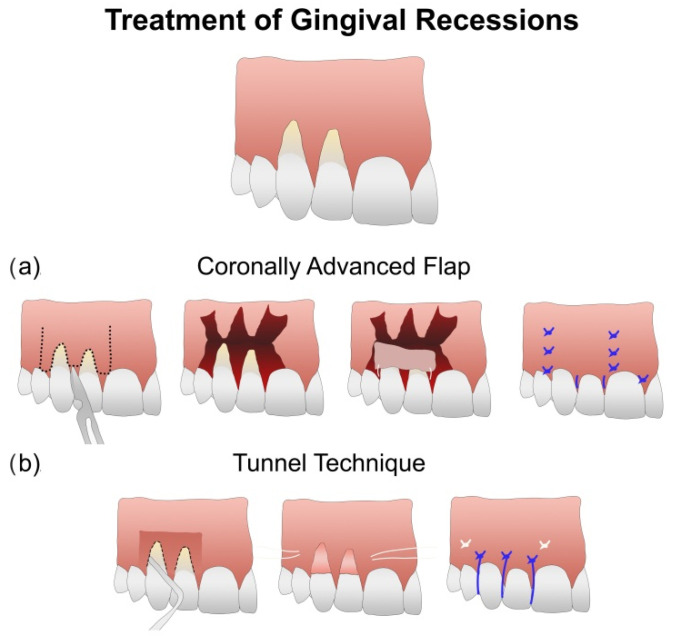
Illustration of the coronally advanced flap (CAF) and tunnel (TUN) surgical techniques for the treatment of gingival recessions. (**a**) CAF involves a flap elevation combining full and split thicknesses followed by the application of a connective tissue graft (CTG) or a polymeric substitute (PolS), which are then sutured over the root surfaces. (**b**) TUN consists of creating a supraperiosteal “envelope” or “pouch” at the gingival margins, allowing flap elevation and insertion of a connective tissue graft CTG or a PolS.

The ultimate goal of periodontal recession treatment is to cover the recession defect. Thus, most efforts are focused on developing techniques that provide root coverage [12]. Gingival recession treatment can be considered a multifactorial treatment approach comprising different surgical techniques and graft materials [12]. In order to obtain complete root coverage (CRC), two different surgery techniques are the most frequently employed: the coronal advanced flap (CAF) [13,14,15,16] and the tunnel technique (TUN) [17,18] (Figure 1). Within the range of adjunctive scaffolds or graft materials available, a connective tissue graft (CTG) is considered the gold standard [19,20,21]. It seems that a CTG may act as a biological filler, improving the adaptation and the stability of the flap to the roots of the teeth. Therefore, a high probability of achieving complete root coverage together with increased soft tissue thickness has previously been attributed to CTG-based approaches [20]. Polymeric substitutes (PolS) have also been proposed [21,22]. These polymeric materials are usually biological and degradable [21,22], and provide a variable, porous surface with an interconnected surface area for direct tissue regeneration [23].

It is worth noting that a CTG is usually acquired from the palatal mucosa, and pain, inflammation and, sometimes, infection have been reported as secondary adverse effects for the patient [4,19,20].

Periodontal plastic procedures are complex, technique-sensitive interventions, so it is important to identify which ones produce the best results [24]. Several studies have compared the efficacy of TUN and CAF + CTG without finding any statistical differences between the two techniques [25,26,27,28]. Currently, new clinical studies are being carried out with a longer follow-up, which could help clarify the clinical differences between the two techniques.

The objective of this systematic review and meta-analysis was to compare the CAF and TUN techniques in conjunction with CTG or PolS to establish which one provides the most effective root coverage for the treatment of unitary or multiple gingival recessions by reviewing the existing literature.

## 2. Materials and Methods

### 2.1. Study Registration and Protocol Development

Before conducting this study, a proposal for this systematic review focused on the coronally advanced flap technique versus the tunnel technique for gingival recession treatment was registered with the International Prospective Register of Systematic Reviews (PROSPERO), and an identification number was given (CRD42021258170). Likewise, the systematic review was executed according to PRISMA-P [28], following the Cochrane Handbook for Systematic Reviews of Interventions [29] and using the PRISMA [30] checklist to increase the transparency and quality of the review.

### 2.2. PICO Question and Focused Question

○ Population (P): Patients presenting single or multiple gingival recessions classified as Miller I, II or III or RT1 or RT2 with an indication of mucogingival surgical treatment (esthetic requirements, hypersensitivity, soft tissue deficiencies).
○ Intervention (I): Gingival recessions (single or multiple) treated with the coronally advanced flap technique at periodontal sites.
○ Comparison (C): Gingival recessions (single or multiple) treated with the tunnel technique at periodontal sites.
○ Outcome (O): Percentage of root coverage.

The focused question is: in patients requiring mucogingival surgery, for unitary or multiple recessions, would it be more effective, in terms of the percentage of root coverage, to perform a CAF or a TUN technique when using a CTG or a PolS?

### 2.3. Information Sources and Screening Process

Two researchers (M.T.-O. and E.M.-S.) performed an electronic search including studies published up to May 2021 in four online databases: the National Library of Medicine (MEDLINE by PubMed), the Cochrane Oral Health Group Trials Register, EMBASE and Web of Science (WOS). A filter was applied in order to search only for articles written in English. Electronic databases and search strategies are provided in Table 1. Additionally, a hand search was conducted to identify manuscripts in previous reviews comparing these two mucogingival techniques for the treatment of gingival recessions. The searches were updated in October 2021.

### 2.4. Eligibility Criteria

Articles meeting the following criteria were included in our systematic review: (1) randomized clinical trials (RCT) or controlled clinical trials (CCT); (2) adult subjects with single or multiple gingival recessions treated with the coronally advanced flap technique compared to those treated with the tunnel technique and using a CTG or a PolS; (3) a follow-up period of at least 4 months; (4) outcomes reported as a percentage of root coverage. Manuscripts not meeting these study criteria were excluded. In addition, some exclusion criteria were also established: (1) in vitro and pre-clinical studies, cohort studies and systematic reviews; (2) studies with a less than 10 patients; (3) mucogingival surgeries combined with bone regeneration techniques; (4) no clinical outcomes clearly reported.

### 2.5. Study Selection, Data Extraction and Assessment of Risk of Bias

Two independent researchers (M.T.-O. and E.M.-S.) performed the study selection. In case of disagreements, it was discussed with a third researcher (M.T.) who determined the inclusion or exclusion of the study.

Data extraction was also performed by two investigators (M.T.-O. and E.M.-S.) and the following information was obtained: (1) authors; (2) year of publication; (3) study design; (4) follow-up time; (5) type of gingival recessions; (6) surgical technique and patients; (7) primary outcome (percentage of root coverage); (8) secondary outcomes (complete root coverage, CRC; width keratinized tissue gain, KTW; red esthetic score, RES).

The risk of bias in the included RCTs was evaluated following the Cochrane Collaboration’s tool, RoB 2 [31]. After analyzing different domains of bias, studies were classified as “high risk”, “some concerns” or “low risk”. The ROBINS-I tool [32], with specified bias domains, was employed for the assessment of the CCTs. Studies were judged as having a “critical risk of bias”, “serious risk of bias”, “moderate risk of bias” or “low risk of bias”.

### 2.6. Data Analysis

A statistical analysis was performed to compare the efficacy of the coronally advanced flap technique with the tunnel technique in terms of root coverage (%) at time points ranging from 4 to 12 months. Weighted means with a 95% confidence interval (CI) were calculated, including the inverse variance (IV) and total sample size. In addition, a subgroups analysis was performed in order to compare multiple vs. single recession and connective tissue graft vs. polymeric substitutes. A subgroup analysis was also employed in order to analyze the effect of both techniques at 6 and 12 months independently. An inter-group analysis was performed in order to compare CTG with PolS when combined with the TUN or CF technique. The variation, or heterogeneity, across the included studies was assessed using the Higgins (I^2^) statistic. Due to the existing heterogeneity from combining the studies, a random-effects model was applied in order to analyze effect sizes. Statistical significance was set at 0.05. RevMan 5.4 (The Cochrane Collaboration, Oxford, UK) was used for data analysis and to produce the funnel plots for representing publication bias and heterogeneity among the included studies.

Ad hoc Galbraith [33] and Baujat [34] plots were constructed using Stata 16.1 (Stata Corp, College Station, TX, USA) in order to explore the relative contribution of each primary-level study to overall heterogeneity in more depth and to control for the presence of potential outliers.

## 3. Results

### 3.1. Search Results

**Figure 2:**
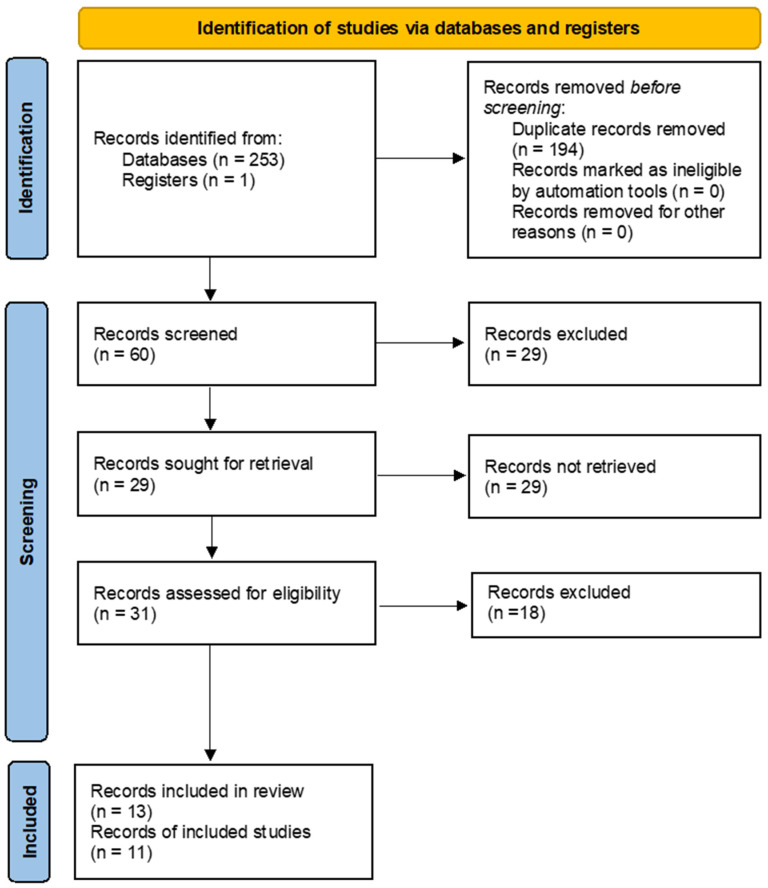
Flow diagram of the present systematic review and meta-analysis following PRISMA guidelines.

A total of 253 articles were obtained after conducting the electronic search. These findings were complemented with a manual search, resulting in one more article. After the duplicates were removed, the title and abstract of 60 articles were read, and the full text of 31 articles was reviewed. Eighteen articles were excluded following the defined eligibility criteria. Finally, 13 articles were included in this systematic review and 11 of them were used for the meta-analysis. Two studies were excluded from the meta-analysis for the following reasons: (1) Zuhr et al., 2021 [35] because the study groups were not comparable; (2) Bhatavadekar et al., 2019 [36] because of incomplete data reporting. The inclusion process based on the PRISMA guidelines is reported in Figure 2.

### 3.2. Study Characteristics

Twelve studies included in the review were RCTs, and only one was a CCT. Each study compared the CAF technique with the TUN technique, eight with a connective tissue graft (CTG) [25,26,36,37,38,39,40,41], four with a PolS (acellular dermal matrix) [42,43,44,45] and one study employed the CAF technique with an enamel matrix derivative (EDM) as the test group and the TUN technique with a CTG as the control group [45]. The main characteristics of the studies included in this systematic review and meta-analysis are displayed in Table 2. From the thirteen articles included in this systematic review, eight of them specified that the adults had been older than 18 years; one study included patients over 16 years old; and the other four studies did not clarify this point, simply reporting that the patients were adults.

### 3.3. Quality Assessment and Risk of Bias

**Figure 3:**
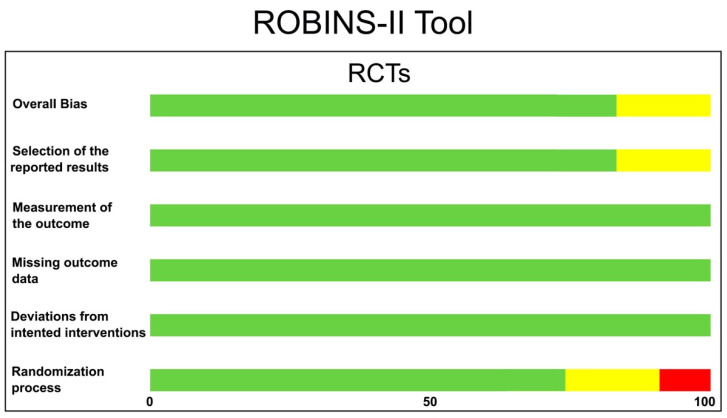
Quality evaluation of the RCTs using the RoB 2 tool (Cochrane Collaboration). The risk of bias in the included studies was classified as either low (green), some concerns (yellow) or high (red).

**Figure 4:**
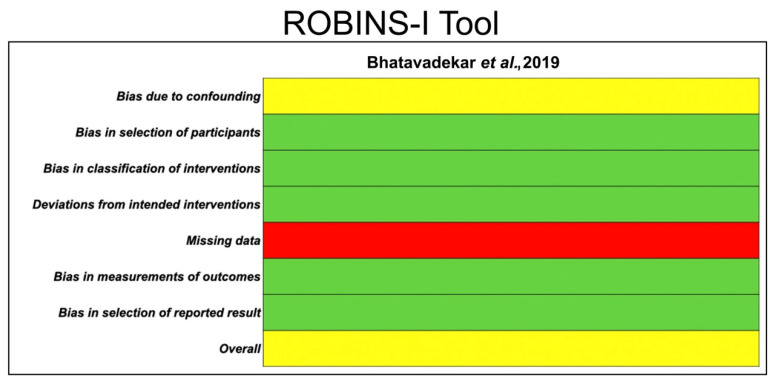
Quality evaluation of the CCT using the ROBINS-I tool. The risk of bias in the included study was classified as low (green), moderate (yellow) or serious (red).

The quality evaluation of the included studies, and the risk of bias assessment using ROBBINS-II (Cochrane recommendations) for the RCTs and ROBINS-I for the CCT, are shown in Figure 3 and Figure 4, respectively.

### 3.4. Primary and Secondary Outcomes

**Figure 5:**
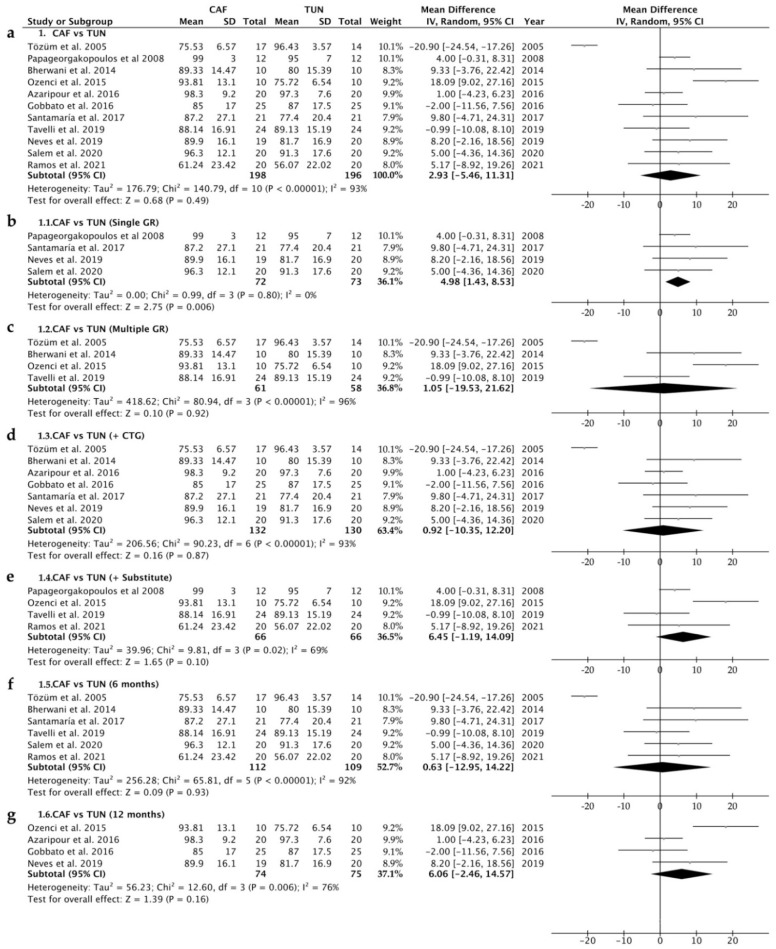
Forest plot for TUN (control group) versus CAF (test group) when comparing the percentage of root coverage after surgical treatment for: (**a**) single or multiple GRs and the use of a connective tissue graft or a polymeric substitute; (**b**) single GRs and the use of a connective tissue graft or a substitute; (**c**) multiple GRs and the use of a connective tissue graft or a polymeric substitute; (**d**) single or multiple GRs and the use of a connective tissue graft; (**e**) single or multiple GRs and the use of a polymeric substitute; (**f**) single or multiple GRs and a 6 month follow-up; (**g**) single or multiple GRs and a 12 month follow-up. The weighted means are presented with a CI of 95%. Heterogeneity was determined using Higgins (I^2^). A random-effects model was applied to all analyses. Statistical significance was set at 0.05.

A total of 394 patients with single or multiple gingival recessions were surgically treated with the CAF technique (198 patients) or the TUN technique (196 patients). When the CAF group was compared to the TUN group, the percentage of root coverage was similar for both groups (*p* = 0.49) with a mean difference of 2.93% (ranging from −5.46 to 11.31; 95% CI). The heterogeneity was I^2^ = 93% (Figure 5a).

When comparing these two techniques separately for single and multiple gingival recessions, the root coverage was significantly higher for the CAF group when treating single recessions (*p* = 0.006) with a mean difference of 4.98% (ranging from 1.43 to 8.53; 95% CI) (Figure 5b). A non-significant difference was found for multiple recessions (mean difference: 1.05%; ranging from −19.53 to 21.62; 95% CI) (Figure 5c).

Similarly, there were no differences between the two surgical techniques when either a connective tissue graft or a PolS was applied. The mean difference between the percentage of root coverage was 0.92% (ranging from −10.35 to 12.20; 95% CI) (Figure 5d) and 6.45% (ranging from −1.19 to 14.09; 95% CI) (Figure 5e) for CTG and PolS, respectively.

**Figure 6:**
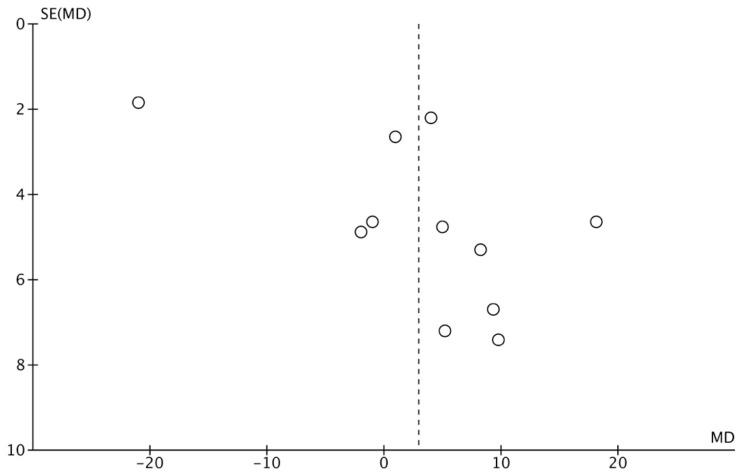
Funnel plot graph illustrating the publication bias and the systematic heterogeneity of the included studies. The standard error (SE) is represented on the vertical axis and the percentage of root coverage (MD) on the horizontal axis.

A random-effects model was applied to all of the analyses performed. The systematic heterogeneity of the included studies is represented in a funnel plot graph (Figure 6).

**Figure 7:**
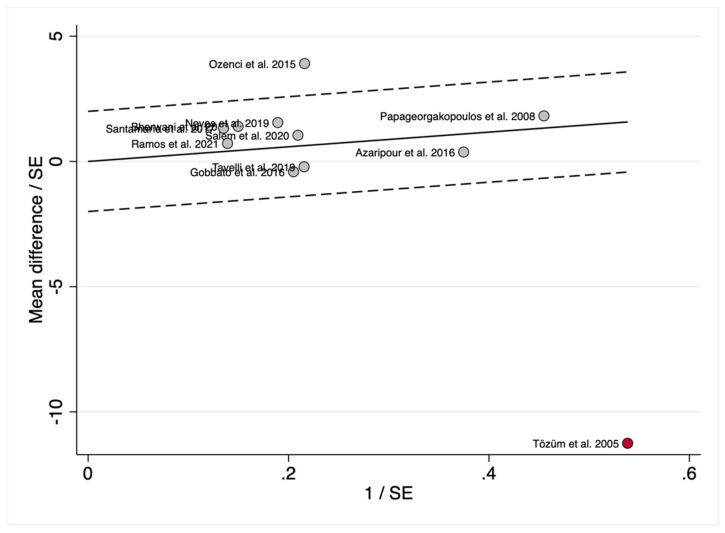
Galbraith plot for TUN (control group) versus CAF (test group) when comparing the percentage of root coverage after surgical treatment, constructed to examine the contribution of individual studies to the heterogeneity metrics and identify outliers. The vertical axis represents the observed effect sizes standardized according to their corresponding standard errors (y = MD/SE[MD]) against precision on the horizontal axis (x = 1/SE[MD]). The regression diagonal line is projected from the origin (0, 0), and the approximate 95% confidence intervals run between the two intermittent parallel lines at ±2 units above and below the regression line. The ID labels were also included, allowing an easier identification of the studies. The study below the confidence limit was identified as an important outlier (Tozüm et al. [41], depicted as a red circle) that contributed disproportionately to the observed heterogeneity. Abbreviations: CAF, coronally advanced flap; MD, mean difference; SE, standard error; TUN, tunnel.

**Figure 8:**
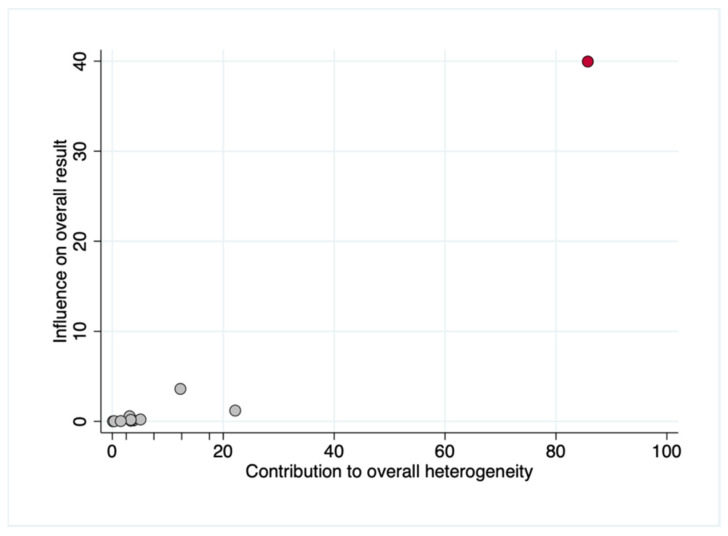
Baujat plot depicting the contribution of individual studies to overall heterogeneity on the *x*-axis (i.e., relative contribution to the Q-statistic) plotted against influence on the overall result on the *y*-axis (leave-one-out method). A Baujat plot was applied to identify the specific subsamples sharing potential sources of heterogeneity. The most heterogeneous and influential primary-level studies should appear in the upper right area of the graph. ID labels were included to allow easier identification of the studies. This graphical method allowed us to identify that Tozüm et al. [41] (depicted as a red circle) provided the greatest contribution to heterogeneity, and could potentially harbor differential clinical, methodological or statistical characteristics as the main source of heterogeneity.

**Figure 9:**
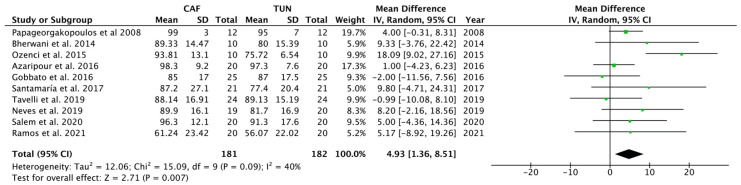
Forest plot for TUN (control group) versus CAF (test group) when comparing the percentage of root coverage after surgical treatment after excluding Tozüm et al. [41] from the analysis. The weighted means are presented with a CI of 95%. Heterogeneity was determined using Higgins (I^2^). A random-effects model was applied to all analyses. Statistical significance was set at 0.05.

**Figure 10:**
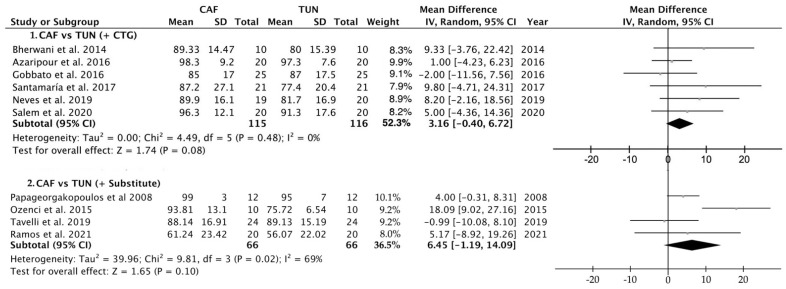
Forest plot for TUN (control group) versus CAF (test group) when comparing the percentage of root coverage after surgical treatment after excluding Tozüm et al. [41] from the analysis. Single and multiple GRs were considered together when using a connective tissue graft or a polymeric substitute. The weighted means are presented with a CI of 95%. Heterogeneity was determined using Higgins (I^2^). A random-effects model was applied to all analyses. Statistical significance was set at 0.05.

Galbraith (Figure 7) and Baujat (Figure 8) plots showed that Tozüm et al. [41] has to be considered as an important outlier. This primary-level study disproportionately contributed to overall heterogeneity, since it was the most influential primary-level study in our meta-analysis. The most relevant salient characteristic of this study was its publication date (i.e., 2005). If Tozüm et al. [41] is excluded from the analysis, two main results are encountered: (i) when comparing the percentage of root coverage attained with TUN vs. CAF, the root coverage was significantly higher (about 4.93%) for the CAF group (*p* = 0.007)(Figure 9); (ii) after inter-group analysis, it is evidenced that there was no significant difference in the percentage of root coverage when using CTG or PolS (*p* = 0.445), regardless of the surgical technique employed (Figure 10).

Regarding the secondary outcomes, CRC was the only outcome investigated by all of the studies in this review. Only Zuhr et al. [35] and Gobbato et al. [40] reported higher frequency of CRC for TUN than CAF. The rest of the studies included in this systematic review found a superior rate of CRC for CAF (100% to 52.6%) than TUN (89.5% to 25%). Regarding KTW, only seven studies examined this parameter. Six of them [25,35,38,39,40,45] declared a higher KTW for the TUN group (2.6 mm to 0.33 mm) compared to the CAF (1.68 mm to −0.36 mm) group. Only Ozenci et al. [42] reported that KTW was higher for the CAF group (1.25 mm) than the TUN (0.87 mm) group. The RES was evaluated by five of the included studies. Zuhr et al. [35] found superior results for TUN (9.06) than CAF (6.94); in contrast, the rest of these studies [25,38,39,42] reported lower esthetic results for the TUN technique (9.3–7.3) than the CAF technique (9.3–8.3).

## 4. Discussion

Over the last decades, numerous periodontal plastic surgery techniques have been proposed for treating gingival recessions [37,46]. The main objective of these procedures is to obtain complete root coverage with optimal integration and acceptable esthetics [17]. Root coverage technique indications can vary depending on whether the recession is single or multiple [18]. The coronal advancement flap technique with posterior modifications [13,14,15,16] is the most documented approach in the scientific literature [47]. In combination with a connective tissue graft, CAF has been considered the gold standard in the treatment of single recessions [48]. In addition, conventional CAF with and without vertical releasing incisions has been successfully used to treat multiple gingival recessions [49]. However, some anatomical conditions may limit its application, such as an insufficient amount of keratinized tissue, non-carious cervical lesions or a reduced vestibule depth [50]. These drawbacks indicate the need for further investigations in the search for alternative approaches.

Raetzke [51] proved in 1985 that envelope/tunnel techniques are an effective alternative treatment in root coverage for unitary and multiple recessions [4]. The tunnel technique is proposed as a minimally invasive, safe and predictable technique [42]. This approach consists of creating a partial thickness “envelope” allowing flap elevation and insertion of a CTG or a polymeric substitute without detachment of the papillary tissues and without vertical releasing incisions [17,52]. TUN has slowly gained in popularity as an option that does not affect the continuity of the interdental papilla in addition to better esthetic results, undisturbed blood supply and nutrition of the graft and a limited flap opening [18]. All of these advantages may result in faster healing and less postoperative morbidity [18,52].

The present systematic review and meta-analysis was designed to shed light to some yet unsolved clinical questions. The first one was a comparison of the effectiveness in terms of percentage of root coverage of the two different surgical techniques (CAF or TUN) using CTG or PolS when treating unitary or multiple gingival recessions. Several systematic reviews [4,47,53] have already studied and compared these techniques without reaching a common consensus or providing evidence on which technique produces a better clinical outcome. This lack of evidence may be due to the limited number of RCTs comparing the two techniques or the absence of a standardized surgical protocol among the clinical researchers. The protocols executed are quite heterogeneous in terms of variation in techniques and the soft tissue substitutes employed. This fact also contributes to the previously mentioned discrepancy. The heterogeneity in the literature regarding this topic may also be explained by the center effect and the surgeon’s ability in such a sensitive procedure [4,21,54].

From the reviewed literature, thirteen studies met the established eligible criteria to be included in our systematic review. Twelve were RCTs and only one was a CCT. Two RCTs [35,36] could not be included in the meta-analysis for not using a tissue substitute in the test group and not having SD available (Table 2). Thus, only ten RCTs and one CCT were included in the meta-analysis.

In the light of the results of our systematic review and meta-analysis, which included 394 patients, it can be stated that both techniques can be considered predictable. The outcomes in terms of root coverage ranged 61.24–99% and 56.07–97.3% for CAF and TUN, respectively. The difference between the two techniques, 2.93% (−5.46 to 11.31; 95% CI), though in favor of CAF, was not significant. Nevertheless, as shown by the Galbraith plot (Figure 7), the study by Tozüm et al. [41], which was the most influential primary-level study in our meta-analysis, should be considered an important outlier. This was also demonstrated by a Baujat analysis, as shown in Figure 8. For this reason, another meta-analysis was performed excluding Tozüm et al. [41]. The results, displayed in Figure 9, show that in this case CAF was superior to TUN (*p* = 0.007), and the difference in the percentage of root coverage was 4.93% (1.36 to 8.51; 95% CI).

For most of the secondary outcomes studied, differences in the absolute values reported by the studies were encountered, but not all of them were statistically significant. It was clear for CRC since most of the studies reported significant differences (double-fold) in favor of CAF with the exception of Zhur et al. [35], which found a higher frequency of CRC when using the TUN technique at all timepoints studied. In the case of KTW, there is still controversy. Only Gobbato et al. [40] and Ozenci et al. [42] found significant, but contradictory, differences. The first group found the TUN technique was superior (*p* = 0.002), whereas the second group was in favor of the CAF technique (*p* = 0.027). Regarding RES, only Ozenci et al. [42] reported a statistically significant difference, which in this case, was in favor of CAF (*p* = 0.034). There is not a clear explanation for these discrepancies. However, in general, it has been shown that the CAF technique may be considered as having superior results for these tested secondary outcomes.

Our subgroup analysis revealed that there was clear evidence for the benefits of using CAF instead of TUN when treating a single GR (*p* = 0.006). This result is in accordance with the suggestion made by Zuhr et al. [55], that discouraged TUN and recommended CAF, leaving the coronal part of the CTG exposed for GR deeper than 5mm [4]. One of the greatest advantages of the TUN technique is the mobility gained by the flap after tunneling the GRs. This does not occur when a single GR is treated, which reduces the efficacy of this technique, and may explain the primacy of CAF over TUN when treating single GRs.

The beneficial effect of applying a graft to treat GR is undeniable, however the question about the use of polymeric tissue substitutes in conjunction with either technique has yet to be answered. Bhatia et al., in a recent meta-analysis [56], showed that combining a CAF with a connective tissue graft or a PolS (collagen matrix) had a significantly higher probability of complete root coverage than a CAF alone. These results were supported by the findings of other authors [47,57]. From our results, soft tissue polymeric substitutes are a valid alternative to CTG (*p* = 0.445 after inter-group analysis). PolS have showed good clinical behavior, achieving a gain in gingival thickness and keratinized mucosa that is very similar to CTG. Palatal harvesting techniques play a crucial role in soft tissue augmentation procedures, but they are not free from disadvantages and complications [4,19,21]. An autogenous graft requires a donor area that will heal by secondary intention, increasing patient morbidity in terms of post-operative discomfort and procedural time [4,58]. In addition, graft harvesting may increase the risk of postoperative complications such as bleeding due to damage of the branches of the palatine artery, necrosis of the mucosa and hypo- or an-esthesia [19,59]. Therefore, the use of PolS should be encouraged.

In their last systematic review and meta-analysis, Cairo et al. [60] confirmed that CTG provided better results than a soft tissue substitute only in regards to some esthetic outcomes. They did not find differences in the esthetic results between CAF or TUN; thus, they suggested that the most likely effect of the grafting material on the esthetic aspect is related to something other than the flap design. This fact was also highlighted by Tavelli et al. [61] when comparing CAF vs. TUN in terms of the root coverage esthetic score.

Among the studies included in the present systematic review and meta-analysis, only three reported on the use of microsurgery [25,40,44]. Microsurgery has been claimed to facilitate complete root coverage in previous reports [62]. It can be observed in our modified Forest Plot (Figure 9) that the two studies [25,44] with the highest weights in the meta-analysis used microsurgery, meaning they provided both strong evidence and low variance in their results. These findings are, therefore, in line with the previously mentioned published data [62].

This study presents certain limitations. Firstly, the focus of the question was narrow, which is, in general, a drawback of systematic reviews. Secondly, the heterogeneity of our study was I^2^ = 93%, which is a relatively high value that may be due to the small sample sizes of the RCTs included in the meta-analysis. This effect has been named the “small-studies effect” [63,64]. However, it should be recognized that most of them were classified as having a low risk of bias. It is remarkable that when we repeated the meta-analysis without the outlier study [41], the heterogeneity of the studies was reduced to 40% (Figure 9). This is one of the more obvious examples of the outlier effect exerted in this study during the first analysis.

For these reasons, it should be necessary that researchers and clinicians make an effort to increase the number of RCTs regarding soft tissue surgeries and boost multicentric studies in order to increase the number of patients in those RCTs. It is also recommended that common protocols, follow-up periods and measured outcomes are established in order to improve the comparability of clinical studies. In addition, the CONSORT guidelines should be followed to lower the risk of bias in clinical trials. This should improve the quality of future systematic reviews and meta-analyses, increase the available evidence, and ultimately, upgrade the standard and the predictability of our treatments.

## 5. Conclusions

Considering the limitations of the present study, it can be concluded that there is some evidence indicating that CAF is more effective than TUN in terms of the percentage of root coverage. There are clear benefits in using CAF instead of TUN when treating a single GR. Both autograft and polymeric substitutes were equally effective when employed with any of the two tested surgical procedures.

## Figures and Tables

**Table 1:** Electronic databases and search strategies.

Keywords	Databases
#1 tunnel technique OR TUN OR pouch OR coronally advanced flap modification tunnel technique OR coronally advanced tunnel technique OR coronally advanced tunnel flap OR tunneling technique OR tunneling flap OR coronally positioned tunnel OR MCAT	PUBMED
#2 gingival recession * OR recession *	Cochrane Library
#3 collagen matrix OR xenogenic collagen matrix OR acellular dermal matrix OR porcine collagen matrix OR porcine derived acellular dermal matrix OR dermal substitute OR polymeric membranes OR polymeric membrane OR connective tissue graft	(CENTRAL)
	EMBASE
#1 AND #2 AND #3	WOS

**Table 2:** Main characteristics of the included studies investigating the surgical technique used for gingival recession treatment with the primary outcome.

Author	Study Design	Follow-Up	Type of GR	Surgical Technique (Patients)	% Root Coverage Mean (SD)
Ramos et al., 2021 [45]	RCT	6 months	Single and multiple	CAF + PolS (20) TUN + PolS (20)	61.24 (23.42) 56.07 (22.02)
Zuhr et al., 2021 [35]	RCT	12 months	Single and multiple	CAF + EMD (13) TUN + CTG (15)	71.8 (20.3) 98.4 (3.6)
Salem et al., 2020 [37]	RCT	6 months	Single	CAF + CTG (20) TUN + CTG (20)	96.3 (12.1) 91.3 (17.6)
Tavelli et al., 2019 [43]	RCT	6 months	Multiple	CAF + PolS (24) TUN + PolS (24)	88.14 (16.91) 89.13 (15.19)
Bhatavadekar et al., 2019 [36]	CCT	12 months	Multiple	CAF + CTG (21) TUN + CTG (15)	96.90 (NR) 89.56 (NR)
Neves et al., 2019 [38]	RCT	12 months	Single	CAF + CTG (19) TUN + CTG (20)	89.9 (16.1) 81.7 (16.9)
Santamaría et al., 2017 [39]	RCT	6 months	Single	CAF + CTG (21) TUN + CTG (21)	87.2 (27.1) 77.4 (20.4)
Azaripour et al., 2016 [25]	RCT	12 months	Single and multiple	CAF + CTG (20) TUN + CTG (20)	98.3 (9.2) 97.3 (7.6)
Gobbato et al., 2016 [40]	RCT	12 months	Single and multiple	CAF + CTG (25) TUN + CTG (25)	85 (17) 87 (17.5)
Ozenci et al., 2015 [42]	RCT	12 months	Multiple	CAF + PolS (10) TUN + PolS (10)	93.81 (13.10) 75.72 (6.54)
Bherwani et al., 2014 [26]	RCT	6 months	Multiple	CAF + CTG (10) TUN + CTG (10)	89.33 (14.47) 80.00 (15.39)
Papageorgakopoulos et al., 2008 [44]	RCT	4 months	Single	CAF + PolS (12) TUN + PolS (12)	99 (3) 95 (7)
Tözüm et al., 2005 [41]	RCT	6 months	Multiple	CAF + CTG (17) TUN + CTG (14)	75.53 (6.57) 96.43 (3.57)

PolS, polymeric substitute; CAF, coronally advanced flap; CCT, controlled clinical trial; CTG, connective tissue graft; EMD, enamel matrix derivative; GR, gingival recession; NR, not reported; RCT, randomized clinical trial; SD, standard deviation; TUN, tunnel.

## Data Availability

The data presented in this study are available on request from the corresponding author.
